# Clinical consensus regarding the importance of rapid reduction in depressive symptoms in major depressive disorder with acute suicidal ideation or behavior (MDSI)

**DOI:** 10.1192/j.eurpsy.2022.1428

**Published:** 2022-09-01

**Authors:** S. Borentain, A. Nash, E. Daly, K. Joshi, M. O’Hara, Q. Zhang, M. Mathews, S. Haughey, S. Richards, J. Anjo, D. Zante, R. Perry

**Affiliations:** 1 Janssen, Global Services Llc, Titusville, United States of America; 2 Janssen, Scientific Affairs Llc, Titusville, United States of America; 3 Janssen, Emea, High Wycombe, United Kingdom; 4 Farmaceutica, Janssen-cilag, Pedroso, Portugal; 5 Janssen Inc, Health Economics & Reimbursement, Toronto, Canada; 6 Adelphi Values, Prove, Bollington, United Kingdom

**Keywords:** esketamine, suicidal behavior, major depressive disorder, suicidal ideation

## Abstract

**Introduction:**

Patients with major depressive disorder (MDD) with acute suicidal ideation or behavior (MDSI) require immediate intervention. Though oral antidepressants can be effective at reducing depressive symptoms, they can take 4–6 weeks to reach full effect.

**Objectives:**

This study aimed to identify unmet needs in the treatment of patients with MDSI, specifically exploring the potential clinical benefits of rapid reduction of depressive symptoms.

**Methods:**

A Delphi panel consisting of practicing psychiatrists (n=12) from the US, Canada and EU was conducted between December 2020–June 2021. Panelists were screened to ensure they had sufficient experience with managing patients with MDD and MDSI. Panelists completed two survey rounds, and a virtual consensus meeting.

**Results:**

This research confirmed current unmet needs in the treatment of patients with MDSI.

Hopelessness, functional impairment, worsening of MDD symptoms, recurrent hospitalization and higher risk of suicide attempt were considered as key consequences of the slow onset of action of oral antidepressants.

Treatment with rapid acting antidepressant was anticipated by panelists to provide short-term benefit such as rapid reduction of core MDD symptoms which may contribute to shorter hospital stays and improved patient engagement/compliance, allowing for earlier interventions and improved patient outcomes. For long-term benefits, panelists agreed that improved daily functioning and increased trust/confidence in treatment options, constitute key benefits of rapid-acting treatments

**Conclusions:**

There is need for rapid-acting treatments which may help address key unmet needs and provide clinically meaningful benefits driven by the rapid relief of depressive symptoms particularly in patients with MDSI.

**Disclosure:**

SB, ED, KJ, MO’H, QZ, MM, MH, SR, JA and DZ are employees of Janssen and hold stock in Johnson & Johnson Inc. AN is currently employed by Neurocrine Biosciences Inc. RP is an employee of Adelphi Values PROVE hired by Janssen.

